# Hospital Meetings, &c.

**Published:** 1898-12-17

**Authors:** 


### HOSPITAL MEETINGS, &C.

### LONDON HOSPITAL.

The customary quarterly general Court of Governors of the London Hospital was held on Wednesday, 7th inst., Mr. J. Hampton Hale presiding. The report presented by the House Committee stated that since the last quarterly court considerable progress had been made with the temporary buildings, and estimates are now being obtained for the warming of the building and the supply of hot water. The temporary photographic department is also well advanced, while the temporary isolation block is complete and occupied. A dispensers' storeroom has also been fitted up, and is found of great use. In accordance with the requirements of the district surveyor, the foundations of the hospital have been exposed at several points and found quite satisfactory. Under favourable circumstances it is hoped to begin the real work on the hospital improvements early in 1899. The extensions of the Medical College approach completion. The college will then be one of the most perfectly equipped schools of medicine in England. For the reception and accommodation of nurses, No. 61, Philpot Street, is being fitted up, and will shortly be ready. The chaplain's department is working satisfactorily, and St. Philip's Church is now quite easy of access to the nursing staff, the arrangements made being much approved of.

The committee are pushing forward the negotiations for the purchase of a site for the new out-patients' department, but unfortunately they are delayed by the obstinacy of one of the lessees, who in the case of five houses holds under them for a term of years, and has sublet for the full period, less five days on four of the houses and twenty-one days in the case of the remaining one. The committee have bought up the sub-leases, but are unable to persuade their lessee to part with his short and quite valueless interest. They are, therefore, obliged to apply for an Act of Parliament, by which it is hoped they will be able to secure this needed interest.

Mr. Eve has been elected to fill the post of surgeon to the hospital left vacant by the resignation, through ill-health, of Mr. McCarthy; Mr. Hutchinson has also been elected surgeon in consequence of the resignation of Mr. Treves; and it was now proposed that Mr. Treves, who has been a member of the surgical staff for a long time with so much honour to himself and to the institution, be elected consulting surgeon to the hospital. Mr. Roxburgh has been elected assistant surgeon, and has also been appointed ophthalmic surgeon to assist Mr. Warren Tay.

The system of payment for medicines and bandages instituted in July last continues to work very satisfactorily, and the fact is gradually becoming more widely known in the East End among those who need to go to the hospital for aid that it s not absolutely necessary to secure a governor's or subscriber's letter before going there for treatment. Patients needing medical attention are seen at whatever hour they may present themselves in the receiving room, and when it is considered necessary by the doctor in charge that they should have further medical attention they are referred to the out-patient department. Letters are, however, still sent out to those of the old governors and subscribers who particularly ask for them, and with them the patients go to the ophthalmic department at the usual hour of one o'clock. But without these letters patients can go to the receiving room to be treated, provided only that they are fit and proper cases for treatment in the hospital.

In moving the adoption of the report, the Chairman desired to add that the private nursing staff had now been increased so as to be in a position to fulfil all calls likely to be made upon it. He also wished to express the regret they felt at Mr. Treves' retirement at a much earlier age than was usual. This was due to the very great increase in his private practice, and he would still, as consulting surgeon, retain his personal interest in the institution. The average number of out-patients treated during the past quarter had been less than in the previous quarter; the in-patients, on the other hand, had been more numerous, as many as 714 having been received on one occasion, while the average had been 673.

The report was adopted *nem. con.*, and a vote of thanks to the chairman brought the proceedings to a close.

### CHARING CROSS HOSPITAL.

THE LORD MAYOR presided yesterday at the Mansion House at a meeting of the Special Appeal Committee of the Charing Cross Hospital. Amongst those present were: The Duke of Norfolk, Lord Glenesk, Sir Henry Burdett, Lieutenant-Colonel and Sheriff Clifford Probyn, Prebendary Kitto (chairman of the Appeal Committee), and Mrs. Kitto, Mr. A. G. Lund (secretary of the Appeal Committee), Mr. T. Percy Borrett, Mr. George Drummond, and Mr. A. E. Reed (secretary of the hospital).

The LORD MAYOR, in opening the proceedings, dwelt upon the encouraging success which had attended the appeal.

Mr. LUND then read a report, in which it was stated that in the two years which had elapsed since the appeal was started, the total amount of donations and subscriptions received and promised was £39,007, and subsequently other donations had been obtained, making altogether £40,619. With this money the council had paid off the heavy debt of the institution of upwards of £17,000, and had spent more than £16,000 in acquiring the freehold of those portions of its premises which would be required to be taken into the hospital in order to make it thoroughly efficient. Every donation which was now given would be available for the much-needed improvements and additions, including new out-patients' department, nursing home, special wards for special cases, and rooms for resident staff. While the appeal committee were grateful for the contributions which had been given during the last two years, they were fully sensible that great efforts must still be made to secure the large amount yet required, and they trusted that the philanthropy of the charitable public would not be wanting to support the movement and so make Charing Cross Hospital thoroughly equipped in every department for its work.

The Duke of NORFOLK, in moving that the report should be adopted and circulated, said that the appeal was in every way worthy of support, and he trusted that the generous City on the east of the hospital and the luxurious district on its west would join hands in providing the money that was asked for. The population of London was growing enormously every year, and with it the claims of the sick and suffering on their fellow men also continued to increase.

Lord GLENESK, who seconded the motion, spoke of the importance of maintaining Charing Cross Hospital on account of its central situation. A few years ago the number of accident cases treated at it each week was 20, but now there were 200 taken to its doors every week. Before another two years had elapsed £60,000 out of the £100,000 asked for would be required. He believed that if the public only understood clearly the object for which the money was wanted they would cheerfully respond to the appeal of the committee.

This motion being agreed to,

Sir HENRY BURDETT next proposed, "That this meeting gratefully acknowledges the liberal support which the special appeal has received, and pledges itself to do all in its power to raise the sum still needed to render Charing Cross Hospital duly efficient for the great work it carries on." He remarked that for some time past the wealthier members of the upper classes had rather stood aloof from helping the hospitals. The list of subscriptions to the Prince of Wales's Fund showed that to be the case. He did not know the reason, although he was told that some of them felt that, as they lived in London only a short time during the year, and as the local claims on them were heavy, the duty of supporting the hospitals of the metropolis should be left to those who resided in London all through the year. The presence with them that day of the Duke of Norfolk and Lord Glenesk, and the assistance they had received from the Duke and Duchess of Sutherland, showed that such views were not universally held by the members of the class to which he had alluded.

Lieutenant-Colonel and Sheriff PROBYN seconded the resolution, which was carried, and the proceedings terminated with a vote of thanks to the Lord Mayor.

